# Human Pluripotent Stem Cell Culture: Current Status, Challenges, and Advancement

**DOI:** 10.1155/2018/7396905

**Published:** 2018-11-22

**Authors:** Sushrut Dakhore, Bhavana Nayer, Kouichi Hasegawa

**Affiliations:** ^1^Institute for Stem Cell Biology and Regenerative Medicine (inStem), National Centre for Biological Sciences (NCBS), Bangalore, India; ^2^Institute for Integrated Cell-Material Sciences (iCeMS), Institute for Advanced Study, Kyoto University, Japan

## Abstract

Over the past two decades, human embryonic stem cells (hESCs) have gained attention due to their pluripotent and proliferative ability which enables production of almost all cell types in the human body *in vitro* and makes them an excellent tool to study human embryogenesis and disease, as well as for drug discovery and cell transplantation therapies. Discovery of human-induced pluripotent stem cells (hiPSCs) further expanded therapeutic applications of human pluripotent stem cells (PSCs). hPSCs provide a stable and unlimited original cell source for producing suitable cells and tissues for downstream applications. Therefore, engineering the environment in which these cells are grown, for stable and quality-controlled hPSC maintenance and production, is one of the key factors governing the success of these applications. hPSCs are maintained in a particular niche using specific cell culture components. Ideally, the culture should be free of xenobiotic components to render hPSCs suitable for therapeutic applications. Substantial efforts have been put to identify effective components, and develop culture conditions and protocols, for their large-scale expansion without compromising on quality. In this review, we discuss different media, their components and functions, including specific requirements to maintain the pluripotent and proliferative ability of hPSCs. Understanding the role of culture components would enable the development of appropriate conditions to promote large-scale, quality-controlled expansion of hPSCs thereby increasing their potential applications.

## 1. Introduction

The quest to understand early embryonic development and the differentiation into mature cell types dates back to the early twentieth century when important experiments described the development of testicular teratocarcinomas in mice [1]. The observation that they were composed of undifferentiated cells of germ cell origin and could give rise to various types of differentiated cells sparked growing interest in the subject. This was followed by the derivation of embryonal carcinoma cells (ECC) from murine teratocarcinomas, which were cultured as embryoid bodies (EBs) and were multipotent [2]. The observation that even single ECCs obtained from a teratocarcinoma had the capacity to grow indefinitely and give rise to multiple cell types gave proof of the existence of individual pluripotent stem cells and opened a unique window into the study of early mammalian development [3]. This discovery that ECCs could be derived from teratocarcinomas, which are tumors induced by the transplantation of implantation-stage mouse embryos to extrauterine sites in histocompatible hosts, inspired researchers to isolate pluripotent cells directly from embryos itself, thus circumventing the need for generating/obtaining teratocarcinomas for pluripotent stem cell isolation. Subsequently, the *in vitro* culture of pluripotent cells was established by successfully isolating the cells from the inner cell mass (ICM) of normal preimplantation mouse blastocysts, and the term “embryonic stem cell” (ESC) was coined [4, 5], thus distinguishing it from teratocarcinoma-derived pluripotent ECCs. These pioneering experiments determined the optimal time point of isolation of pluripotent ESCs from embryos and allowed the development of appropriate culture conditions to maintain ESC lines in their undifferentiated state with indefinite proliferation capacity [4, 6]. Further advances allowing development of nonhuman primate ESC lines [7] eventually led to the breakthrough establishment of hESC lines.

hESCs are derived from the ICM of preimplantation blastocysts and can propagate and retain their pluripotency when grown in proper culture conditions [4, 6]. These cells show undifferentiated morphology, expression of pluripotency markers, unlimited proliferation, and the potential to differentiate into all three embryonic germ layers, even after prolonged culture, while maintaining a normal karyotype. These features have since then become the defining characteristics of PSCs. Following hESCs, an important discovery was the development of induced pluripotent stem cells (iPSCs) by forced expression of transcription factors necessary for reprogramming adult somatic cells into pluripotent cells. This approach bypassed the need of embryos for obtaining pluripotent stem cells, thereby resolving the ethical concerns posed by hESC research [8].

The unique potential of hPSCs to self-renew in culture and give rise to all somatic cell types in the embryo makes them an exciting candidate for cell replacement therapy (CRT) in various diseases such as degenerative disorders and cancer, as well as offers limitless possibilities for understanding early development and establishing *in vitro* disease models. Studies have demonstrated the capability of hPSCs to differentiate into various cell types derived from ectoderm, endoderm, and mesoderm, such as cardiomyocytes, neurons, glia, hepatocytes, pancreatic islet cells, chondrocytes, skeletal myocytes, adipocytes, and endothelial cells. Thus, an unprecedented level of research is directed towards elucidating the factors involved in regulating pluripotency and differentiation. Knowledge of the same can be applied towards recapitulating developmental stages and understanding the mechanisms underlying normal and diseased states. It therefore has wide-ranging applications in advancing drug discovery, regenerative medicine, and gene therapy.

Furthermore, the use of hiPSCs opened up the possibility of autologous CRT, moving us one step closer to the hope of bringing stem cell therapies from the bench to bedside. It is worth noting that hiPSCs share similar characteristics with hESCs in terms of signaling mechanisms, and the culture systems for hiPSCs are similar to those used for hESCs as well.

Recent studies have shown that “pluripotency” exists in different states, depending on the culture condition of hPSCs. Amongst them, two functionally distinct stem cell states have been identified, namely, “naïve” and “primed,” which are similar to mouse ICM cells in preimplantation blastocyst and epiblast layer cells in postimplantation blastocyst, respectively [9–12]. Conventionally, hESC lines have been derived and maintained in a pluripotent state resembling the primed state in a mouse, represented by mouse epiblast stem cells (mEpiSCs) [9, 11]. The same holds true for hiPSC lines that have been reprogrammed using the method first described by Takahashi et al. [8]. Since the discovery of the two pluripotency states, it has been established that mouse hESCs, which are in a “naïve” state, represent an earlier time point than primed EpiSCs, in mouse embryonic development [9–11]. Consequently, a huge body of research has been aimed at optimizing culture conditions for each stage, and recently, “naïve” hESCs have also been generated by toggling conventional hESCs back from the “primed” state [13–15] or by deriving new hESC lines from human six-cell to eight-cell stage embryos, using naïve-state growth conditions from the beginning of derivation itself [15]. Although hPSCs in the naïve-state growth conditions are more unstable than primed-state condition, these led to an increased understanding of the pathways that are active/inactive *in vitro* and how they can be manipulated by growth factor and small molecule supplementation of media, thus underscoring the importance of hPSC culture media all the more.

**Figure 1:**
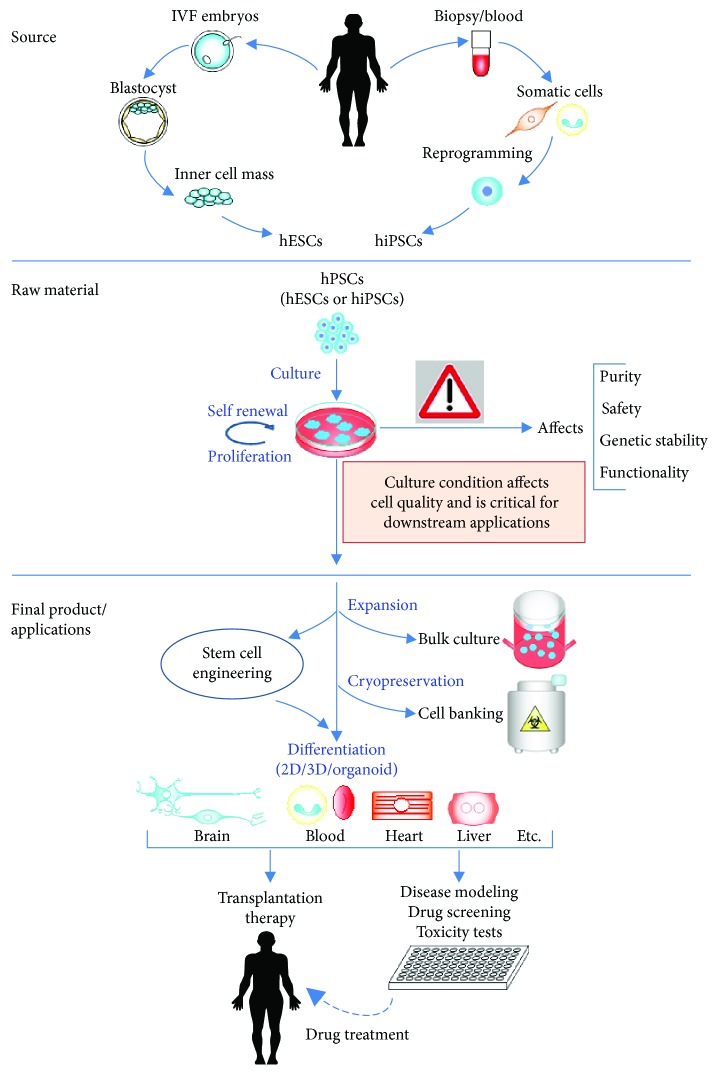
Importance of culture media optimization for stem cell engineering.

The use of hPSCs in all downstream applications requires the establishment of protocols that will allow large-scale, cost-effective cultivation of cells, without compromising on their quality. It is now well known that culture conditions can affect several parameters, which are important to evaluate for stem cell engineering applications, such as gene correction or selection of genetically stable and highly pluripotent populations. Studies have shown how prolonged culture of hPSCs can introduce spontaneous mutations or genomic abnormalities, which invariably affect the purity, consistency, potency, and functional capacity of hPSCs, as they can bias or prime the cells away from their truly pluripotent state [16–21]. Suboptimal hPSC culture conditions can therefore alter their identity and their compatibility with downstream differentiation protocols. This can skew results in research for disease modeling and drug-based studies and also affect the final product that is to be used for transplantation therapy. The possibility of such alterations in hPSC quality and stability, introduced by culture media itself, raises important concerns regarding the safety and risks associated with using these cells for engineering and therapeutic applications (Figure 1).

Since hPSCs are the most powerful and promising raw material for cell engineering applications, understanding and controlling the behavior of hPSCs by optimizing the culture media, small molecules, growth factors, and microenvironment, will allow the identification of appropriate culture conditions for downstream applications such as deriving specific cell types in CRT, drug discovery, and study of human embryogenesis and disease mechanisms. This review describes different media used to maintain hPSCs in their pluripotent state, their components and functions, and advancements in the development of appropriate conditions to promote large-scale, quality-controlled expansion of hPSCs thereby increasing their potential applications.

## 2. Evolution of an Adherent Culture System for hPSCs

### 2.1. Traditional Culture System

The ICM cells, once isolated, need to be placed on suitable appropriate extracellular matrices (ECMs) and cultured in media that can support their pluripotency and maintain self-renewal. Traditionally, the blastocyst-derived cells were plated and serially propagated on mouse embryonic fibroblasts (MEFs) that are mitotically inactivated by mitomycin C treatment or gamma irradiation [6]. Studies that have analyzed fibroblast conditioned media and decellularized matrices with mass spectrometry have revealed that these feeder cells secrete essential growth factors, ECM components, and cytokines into the culture media, which support hESC growth and proliferation, such as fibroblast growth factors (FGFs), transforming growth factor-*β* (TGF*β*), bone morphogenetic proteins (BMPs), Activin A, laminin-511, laminin-binding integrins, vitronectin, heparan sulphate proteoglycans, fibronectin, and collagen [22, 23].

However, several factors can affect the performance of feeder layers, thereby affecting the secretion of factors and deposition of ECM components, which can negatively impact the consistency of feeder-based culture. This would also limit the ability to interpret differences in the biology of hPSCs due to undefined determinants contributed by the feeder microenvironment. Moreover, feeder cells can also be a source of animal pathogens and mycoplasma contamination. In this regard, Martin et al. showed that most animal-derived products were a source of the nonhuman sialic acid Neu5Gc, which get incorporated into hESCs under standard culture conditions, and the transplantation of the same would thus induce an immune response [24]. The use of human feeders was therefore proposed as an alternative to MEFs, in order to prevent the use of nonhuman components. While feeders from fetal human tissue seem to be the most supportive, such as feeders from human fetal muscle and skin [25], fetal foreskin [26], and fetal liver stromal cells [27], the use of aborted embryos for fetal fibroblast extraction poses ethical implications. Therefore, several other human cell types have been tested for their ability to maintain hPSC pluripotency in culture, including human adult fallopian tubal fibroblasts, bone marrow cells [28], umbilical cord [29], placental cells [30, 31], and endometrial cells [32]. Autogenic feeder layers derived from differentiated fibroblasts from the hESCs itself were another approach taken to circumvent the use of donor feeder cells altogether thereby completely eliminating the risk of allogenic pathogen contamination [33, 34]. Despite these innovative advances, it is important to realize the limitation of feeder cell-dependent culture systems, mainly owing to their lot-to-lot variability and inconsistencies between different culture batches, ultimately making them unsuitable for therapeutic applications.

### 2.2. Feeder-Free Culture System

The studies done using different types of feeders, the analysis of the components they produce in culture, and the comparison between different feeder types have enabled us to understand the mechanisms and pathways that are fundamental in maintaining pluripotency. To fully harness the potential of these cells, it is imperative to establish defined culture systems, which can support large-scale generation of hPSCs and their therapeutic derivatives. Since the derivation of hPSCs, several animal-derived products have been employed to provide conditions suitable for maintaining pluripotency and directing them towards differentiation. Traditionally, hPSCs have been cultured on a layer of MEFs in media supplemented with fetal bovine/calf serum (FBS or FCS) and animal-derived growth factors. The use of animal-derived components, however, raises the possibility of xeno-contamination and immune rejection. Hence, these culture systems render hPSCs unsuitable for clinical transplantation. In order to satisfy clinical-grade standards, culture systems that are completely xeno-free need to be developed without compromising on hPSC quality and quantity. Therefore, significant research has been dedicated to understanding the mechanisms that regulate pluripotency. This has led to the development of chemically synthesized xeno-free products and small molecules that allow replacement of all animal-derived components *in vitro*, while maintaining suitable culture conditions for pluripotent stem cells.

Approaches to develop feeder-free systems consisted of identifying a suitable basal medium, as well as a synthetic substrate/coating which would substitute for the defined and undefined soluble factors provided by feeders in culture. For example, Chin et al. identified nearly 30 proteins secreted by feeders derived from different sources, out of which they demonstrated the capability of six proteins in supporting hESC culture [35]. The use of MEF-conditioned media and animal-derived ECM proteins, specifically Matrigel™ (Corning), which was produced by Engelbreth-Holm-Swarm mouse sarcoma cells, or laminin, was reported to stably maintain hESCs for several passages [36]. Since then, several approaches have aimed at replacing both MEF-conditioned media with supplements or small molecules and Matrigel with purified recombinant ECM proteins [22, 37–40]. In the following sections, we discuss the key components of feeder-free media and their roles, an understanding of which is required for the development of chemically defined media.

Attempts to generate defined media for hPSCs, ensuring batch-to-batch consistency, have yielded several commercially available options for feeder-free culture, some of which are xeno-free and some that also comply with cGMP (Current Good Manufacturing Practices) (Table 1). Commonly used commercial media include mTeSR1, TeSR2, TeSR-E8, RSeT and NaïveCult (STEMCELL Technologies), StemPro, Essential 8 and StemFlex (Thermo Fisher Scientific), Pluripro (Cell Guidance Systems), PluriSTEM (Millipore), StemFit (Ajinomoto), and Nutristem (Corning). Some of these media such as TeSR2 contain human serum albumin (HSA), whereas the more recent and widely used Essential 8 medium, which is derived from TeSR2, does not contain either HSA or bovine serum albumin (BSA) and was amongst the first to be considered truly defined media. However, most commercially available and in-house media contain expensive recombinant growth factor proteins, such as FGF2 and TGF*β*/Activin A/Nodal, at varying concentrations, the functions of which will be discussed in upcoming sections.

## 3. Basic hPSC Culture Media Components

### 3.1. Basal Media

DMEM/F12 is the standard basal media used in hPSC culture, and a comparative analysis of 12 different base media did not lead to the identification of any other basal medium which performed better than DMEM/F12 even in serum-free conditions [41]. Apart from glucose, amino acids, and vitamins, which are components of DMEM/F12 itself, cholesterol, lipids, insulin, transferrin, selenium, ascorbic acid, g-aminobutyric acid (GABA), lithium chloride (LiCl), pipecolic acid, and *β*-mercaptoethanol (BME) are other components often included in serum-free culture media. Another component of serum-free media is L-ascorbic acid-2-phosphate, which has been included in media compositions as it enhances hPSC survival and proliferation, along with FGF2, as was shown by Furue et al. and Chen et al. [38, 41]. Cholesterol, another additive, is the precursor of a steroid hormone and a component of signaling proteins, due to which it is also included in media compositions [42]. BME which is often added in media to prevent toxicity from oxygen radicals is itself toxic to cells, and the presence of BSA in media offsets any detrimental effects from BME. Chen et al. showed that in the absence of BME, BSA was no longer necessary for hPSC media [41]. They also reported the need for selenium in media to support sustained expansion and transferrin to improve survival and cloning efficiency [41]. Selenium is also important in culture media as it has been shown to protect cells against oxidative damage by optimizing the activity of glutathione peroxidase and thioredoxin reductase, in addition to stimulating cell growth and proliferation [43].

### 3.2. Microenvironment of hPSC Culture

In general, hPSCs are cultured in normal atmospheric oxygen (21%), although mammalian preimplantation embryos develop in relatively hypoxic conditions in vivo, characterized by 1.5-8% oxygen tension [44]. While hESCs can be maintained reproducibly at 21% oxygen tension, several studies have provided evidence that mimicking physiological oxygen levels (~4%) is beneficial to hESCs by reducing spontaneous differentiation [45, 46] and by upregulation of genes that are known to support pluripotency of hPSCs, suggesting that some transcriptional programs in hPSCs are oxygen-sensitive [47]. In contrast, Chen et al. reported no clear benefit of culturing hESCs in 5% oxygen tension, with respect to morphology, survival, and gene expression, as long as a 7-day splitting interval was maintained [48]. However, others have also reported that low oxygen tension (2%) enhanced hESC clonal recovery and decreased the frequency of spontaneous chromosomal aberrations, without any significant changes in pluripotent marker expression [49]. Furthermore, it has also been shown that 2-5% oxygen tension increases hESC proliferation rate as well as expression of NANOG and POU5F1, the key pluripotency genes [39, 50]. A recent study interestingly reported that hypoxia could indeed influence cell fate decisions in culture, as 2% oxygen alone could reactivate expression of hESC markers in hPSC-derived differentiated cells [51]. These studies show that while hPSCs can be maintained at atmospheric oxygen tension, it may be more beneficial to lower the same to 2–5%; however, the impact is still controversial, and the effects may vary with differences in culture media, splitting intervals, and may be cell line-dependent too.

A study of the physiochemical environment of culture media showed that hESCs were better maintained when the basal media had high D-glucose concentration (4.5 g/l) and an osmolarity that mimicked the natural environment of embryonic tissue, with optimal performance obtained with 320 mOsm [52]. However, the use of 340–350 mOsm has also been reported in the development of TeSR1, mTeSR1, and E8 media [39, 41, 53].

Given the influence that pH has on every biological process and in maintaining homeostasis in vivo and *in vitro*, variations in pH affect several mechanisms within the cells and their microenvironment. The pH of the culture media is an important factor involved in maintenance of hESCs, in the successful reprogramming of somatic cells to hiPSCs, and in the induction of differentiation of hPSCs. Several cellular traits can be affected actively or passively by the pH, such as the motility, enzymatic activity, cell cycle, and apoptosis. It also affects cell motility through changes in the cytoskeletal components [54]. It has also been shown that upon reprogramming, the colonies obtained when the culture media was within pH 7.0 to 7.4 showed a compact morphology with strong alkaline phosphatase activity, whereas a slight change in pH to 7.6–7.8 made the colony morphology dispersed and flat. Similarly, when hPSCs were cultured at different pH values, morphological differences were observed at each pH, with colonies being more compact at a pH of 6.8, and as pH increased to 7.8, the cells were more dispersed and could be observed as single cells.

### 3.3. Serum Alternatives for hPSC Media

As the use of FBS/FCS in hESC cultures was a risk factor, other studies used human serum- (HS-) containing medium and demonstrated the ability of HS to efficiently maintain hESC pluripotency, self-renewal, and a stable karyotype for at least over 30 passages [25, 55]. Although this provided an animal-free alternative, it did not address the issue of undefined serum compositions and lot-to-lot variation, which could lead to variability in hPSC culture with respect to their ability to maintain pluripotency, self-renewal, differential potential, and a stable karyotype. These made them unsuitable for downstream therapeutic applications. One of the earliest attempts to generate a substitute for serum led to the development of the proprietary Knockout Serum Replacement (KSR) [56], now commercially available and used as a standard in media for hPSCs grown on MEFs. Amit et al. showed that the cloning efficiency of hESCs increased by several folds in the presence of 20% KSR as compared to serum-containing medium and that hPSCs derived by serum-containing media could easily be transitioned to KSR-containing media without any compromise on self-renewal capacity or pluripotency [26]. The expression profiles of pluripotency-associated genes were observed to be similar under FBS-containing and KSR-containing media formulations [57]. It was also shown that hPSCs exhibited an increased growth rate when grown in KSR-containing medium compared to FBS-containing medium [58]. Rajala et al. tested several combinations of culture media and showed that KSR-containing medium was superior to HS containing one [59]. Other publications reported the ability of KSR to efficiently derive hPSC lines thus confirming the efficacy of KSR and its compatibility with hPSC media [30, 60].

Further, enormous efforts have been invested in finding essential factors in the serum or serum replacement to come up with a simple and defined culture condition. Supplementation of DMEM/F12 with N2 and/or B-27, in the presence of growth factors like bFGF, was shown to support prolonged self-renewal of hESCs [22, 61]. Replacement of serum and KSR with albumin, specifically HSA and BSA, in TeSR1 [39] and mTeSR1 [53], respectively, has also been widely used for hPSC cultivation. Interestingly, the addition of 0.1% HSA reportedly rescued the loss in hPSC viability caused by insulin depletion in suspension-grown hPSCs, thus demonstrating the diverse ways in which albumin can support hPSC pluripotency and viability in culture [62]. Another serum replacement product, lipid-rich BSA, known as AlbuMAX, was identified as the active ingredient and predominant lipid source in KSR [63]. It is a mixture of albumin with lipids (such as cholesterol, phospholipids, and triacylglycerides) and free fatty acids (alpha-linolenic acid, linoleic acid, oleic acid, stearic acid, and palmitic acid) [64]. It was shown that hESCs could be stably maintained in KSR-deficient media supplemented with 1% AlbuMAX and N2/B27, thus achieving a more defined media composition [63]. Another study showed how supplementation of chemically defined media (CDM) such as E8, with AlbuMAX, reduced the alterations in metabolic flux that are usually induced upon culture in CDM such as E8 [65]. Indeed, most serum replacements include albumin (such as BSA/HSA), as it is the most abundant protein in serum. The addition of albumin helps in protecting the cellular surface and stabilizing other proteins in the culture media, making it a beneficial additive in serum-free media. However, it is not an absolute requirement in hPSC media, as the commercially available media E8 does not include any albumin [41]. In this regard, Chen et al. and Yasuda et al. reported that in the absence of any serum replacement, insulin and transferrin are the two minimum required proteins in addition to growth factors and chemical compounds [41, 66]. Moreover, the purification of albumin from serum or culture supernatants (in the case of recombinant protein expression) can introduce contaminants, owing to albumin's high capacity of binding proteins, ions, chemicals, and pathogens, which are difficult to get rid of upon purification. Further studies are required to elucidate the precise roles and mechanisms by which these proteins in serum replacements maintain pluripotency and self-renewal, so that they can be replaced by defined and cost-effective chemical compounds, which could possibly make serum replacements obsolete in the future.

## 4. Growth Factors for hPSC Culture

### 4.1. Fibroblast Growth Factors (FGFs)

Along with KSR, the earliest identified component that was found to contribute to stem cell pluripotency and self-renewal was basic fibroblast growth factor (bFGF or FGF2), which was shown to be endogenously produced by human feeder layers in hESC cultures [27, 43]. It was found that FGF2 was indispensable for maintaining undifferentiated proliferation of ES cells in KSR-based media [26]. Xu et al. also reported that FGF2 was essential to support undifferentiated hESC growth in serum replacement media, alone or in combination with other growth factors in the absence of feeders or conditioned media, while maintaining comparable morphology, surface marker and transcription factor expressions, karyotype, telomerase activity, and differentiation potential [67]. In feeder-free systems, it has been shown that FGF2 at a range of concentrations (4 to 100 ng/ml) was required to sustain the pluripotency of hPSCs over several passages, equivalent to conditioned media from MEFs [68]. FGF2 was found to stimulate the secretion of supportive factors from MEFs which reduced differentiation-inducing activity, and it regulated the expression of TGF*β* family members, enabling them to act on hPSCs in an autocrine way to promote self-renewal [69]. cDNA microarray analyses of hESCs compared to mESCs and differentiated human cells showed the enrichment of FGF2/13 and FGFR1, 2, and 4 in PSCs [70–72]. Another group validated this with real-time PCR results confirming that FGFR1-4 were indeed expressed in hESCs with FGFR1 being the most abundant [73], while Eiselleova et al. reported that FGF2 dominantly signaled via FGFR2 [74]. Furthermore, addition of the FGFR inhibitor SU5402 led to rapid cell differentiation by suppressing the activation of downstream protein kinases and downregulating OCT3/4, thus suggesting the existence of autocrine FGF signaling in hPSC cultures [73]. Consistent with this, another report showed that the knockdown of FGF2 led to rapid differentiation in hPSCs, and the undifferentiated phenotype could not be rescued by the addition of exogenous FGF2 either. Instead, exogenous FGF2 functioned to reinforce the pluripotency maintenance program of intracrine FGF2 signaling [74]. Altogether, FGF2 promotes hPSC self-renewal and proliferation in the undifferentiated state in several ways. It binds to FGFR and activates the cascade of the mitogen-activated protein kinase (MAPK) pathway, as well as the phosphatidylinositide 3-kinases (PI3-kinase)/AKT pathway, leading to high basal levels of extracellular signal-regulated kinases (ERK) 1/2 and protein kinase B (PKB)/AKT, respectively, in hPSCs, both of which are implicated in the expression of stem cell genes and suppression of cell death and apoptosis genes [73–75]. This was confirmed by Li et al. who also showed that both mitogen-activated protein kinase (MEK)/ERK and PI3K/AKT signalings were downstream targets of the FGF pathway, as shown by high levels of phosphorylated MEKs, ERKs, and PKB/AKT, upon FGF2 treatment of hESCs [75]. Interestingly, while the inhibition of MEK/ERK and PI3K/AKT alone or together led to the rapid loss of self-renewal capacity in hPSCs, the inhibition of only PI3K/AKT led to a significant decrease in cell proliferation and a marked increase in apoptosis, thus suggesting both common and distinct roles of these two pathways downstream of FGF signaling in hPSCs [75]. In another study, it was also shown that neurotrophins (brain-derived neurotrophic factor, neurotrophin 3, and neurotrophin 4) improve hESC survival significantly, and this effect is mediated by the PI3K pathway but not the MEK/ERK pathway [76].

As mentioned above, the MEK/ERK pathway is known as one of FGF target pathways in hPSCs. Kang et al. were amongst the first to report that FGF signaling in hESCs induced the activation of ERK (extracellular signal-regulated kinase), which in turn induced expression of c-fos, an early downstream target of the MEK/ERK1/2 pathway [77]. It was shown that MEK/ERK inhibition in hESCs by specific MEK inhibitors PD98059 and U0126, or by RNA interference, rapidly caused the loss of the undifferentiated state; however, it did not affect cell proliferation or survival, while PI3K/AKT inhibition by LY294002 induced a significant decrease in cell proliferation and increase in apoptosis [75]. These data suggest that in response to FGF, the MEK/ERK pathway supports hPSC self-renewal in cooperation with other pathways such as the PI3K/AKT pathway. The physiological role and targets of MEK/ERK in hPSCs have not yet been clarified and need to be determined. However, as a common effector downstream of both platelet-derived growth factor (PDGF) and FGF, MEK/ERK seems to be crucial for supporting pluripotency-related processes regulated by receptor tyrosine kinases (RTKs).

Heparin and heparan sulfate proteoglycans form high-affinity binding complexes with FGFs and are therefore included in media to control the activity and stability of FGFs [78]. Furue et al. showed that the addition of heparin also promoted hESC proliferation in the absence of FGF2 in a dose-dependent manner [38]. Heparin induced expression of cyclin D1, a cell cycle regulator, and also rapid phosphorylation of the FGF receptors in hESCs, in the absence of exogenous FGF2, suggesting that it helps hPSCs in stabilizing endogenously produced FGF [38]. In addition, heparin has also been found to enhance the activity of Wnt and FGF signalings in hESCs [79].

### 4.2. Other Ligands of Receptor Tyrosine Kinases (RTKs)

A proteomic screen of MEF-conditioned media aimed at identifying candidate growth factors that support hESCs in culture showed that insulin-like growth factor (IGF), specifically IGFII, was the most abundant [80]. It has been shown previously that autologous feeder layers derived from spontaneous differentiation of hESCs into fibroblasts supported the growth of pluripotent hESCs, and these fibroblast-like differentiated hESCs expressed a higher level of FGF receptors than the undifferentiated hESCs in the same culture dish [34]. Under such culture conditions, treatment with FGF2 led to the release of IGFII and TGF*β* factors from the autologous feeder cells which then acted upon the undifferentiated hESCs in a paracrine manner to promote self-renewal and pluripotency [80]. They reported that undifferentiated hESCs expressed IGF1 receptor RTK (IGF1R) which, when blocked, reduced survival and clonogenicity of hESCs, while the surrounding feeder cells expressed FGFR1 which, when blocked, led to differentiation. Their results suggested that IGFII alone could sustain hPSC growth and expansion in long-term culture, and its IGF signaling was mediated via activation of the PI3K/AKT pathway [80]. The importance of insulin in hPSC media was also confirmed by Wang et al. who showed that the IGF1R blocking antibody reduced hESC proliferation and induced differentiation, and moreover, IGF1R-specific shRNA transduced in hESCs was incapable of self-renewal in culture [81]. Other studies aimed at identifying specific factors in serum that promote the growth of hESCs showed that the lysophospholipid sphingosine 1-phosphate (S1P), together with PDGF, needs to be present to maintain hESCs in their undifferentiated state in feeder-free culture and that S1P and PDGF have an antiapoptotic effect in hESCs [82, 83]. Phosphoproteomic analysis of hESCs also revealed that the PDGF receptor RTK (PDGFR) inhibitor led to differentiation of hESCs and a decrease in expression of pluripotency markers. PDGF-AA cooperated with bFGF to stably maintain undifferentiated hESCs in culture, and it helped preserve their undifferentiated state even under suboptimal concentrations of FGF2 [84].

Furthermore, the role of RTK signaling was demonstrated in another study that investigated the contribution of epidermal growth factor receptor (EGFR) family members, in hESC culture. They found that the ERBB2/3 was expressed on hESC cells thus implying a role for its ligand neuregulin 1 (NRG1) [81]. Furthermore, inhibition of ERBB2 significantly reduced hESC proliferation and induced apoptosis in feeder-free cultures [81]. Taking into account the roles of these identified growth factors, Wang et al. assembled a simple feeder-, serum-, and KSR-free defined medium (DC-HAIF), designed to stimulate IGF1R and ERBB2/3 signalings, by incorporating neuregulin 1 (or heregulin-1*β*), FGF2, LR3-IGF1 (a GMP-grade recombinant human IGF1), and Activin A [81]. They showed that hESCs could be successfully and stably propagated in this media for several months with minimal spontaneous differentiation.

### 4.3. TGF*β* Superfamily

The TGF*β* superfamily consists of over 100 proteins, including the TGF*β* proteins, Activin, Nodal, bone morphogenetic proteins (BMPs), and growth and differentiation factors (GDFs), all of which mediate several biological effects through receptor serine/threonine kinases, to maintain stem cell fate. Once activated by binding of TGF*β* family ligands, these receptors phosphorylate and activate Smad proteins, which translocate to the nucleus, and function as transcriptional cofactors to activate target genes. Type I receptors (TGFRI), also termed Activin-like kinases (ALKs), play a central role in pluripotency, as BMP ligands and Activin/Nodal/TGF*β* ligands exert their effects through receptor ALK 2/3/6 (activating SMAD 1/5/8) and ALK 4/5/7 (activating SMAD 2/3), respectively [85].

The effect of eight different growth factors on the differentiation of hESCs was evaluated by Schuldiner et al. wherein TGF*β*1 did not lead to the production of transcripts in differentiated cells, thus suggesting its role in the repression of hPSC differentiation. Amit et al. showed that TGF*β*1 contributed to a cocktail of growth factors that also included FGF2, to maintain the undifferentiated state of hESCs in feeder-free culture [37, 86]. It was reported that in undifferentiated cells, the TGF*β*/Activin/Nodal branch was activated through the signal transducer SMAD2/3 which was achieved by addition of Activin A (25 ng/ml), and this was shown to be required downstream of canonical Wnt activation, which was necessary to maintain hPSCs [87–89]. This dependence was further confirmed by studies which showed that TGF*β*/Activin/Nodal-responsive SMAD2/3 directly binds to the Nanog proximal promoter and activates its expression [90]. The requirement of Activin/Nodal signaling through SMAD2/3 activation for maintaining pluripotency was confirmed by another study as well [88], wherein inhibitors of this signaling pathway led to hPSC differentiation. However, according to this study, neither Nodal nor Activin alone was capable of sustaining long-term hPSC growth, but either of these, when combined with FGF2, helped achieve the optimal conditions for maintaining long-term hPSC pluripotency and self-renewal [88]. This was in agreement with Xu et al. who also showed that either FGF or TGF*β* alone was incapable of maintaining long-term pluripotency of hESCs [91].

While Nodal and Activin A act on the same receptors and activate the same signaling mechanisms to suppress hPSC differentiation [87–89], the latter is used more often *in vitro* for cell culture due to its wider availability as a recombinant protein and comparatively lower cost. Beattie et al. also showed that Activin A was secreted by MEF feeders and enriching the culture medium with a combination of exogenous Activin A (optimum concentration determined to be 50 ng/ml), along with FGF7 (keratinocyte growth factor, 50 ng/ml) and nicotinamide (10 mM), maintained hESCs in an undifferentiated state for over 20 passages without MEF feeders or conditioned medium [92]. Removal of Activin A on the contrary led to rapid differentiation of hPSCs thereby confirming the inevitability of this growth factor in hPSC culture media [92]. A detailed study by Xiao et al. provided evidence for the first time that low concentrations of Activin A (5 ng/ml) was necessary and sufficient to support undifferentiated hESC growth on feeder-free culture (Matrigel) [40]. They also showed that Activin A induced the expression of OCT4, Nanog, Nodal, Wnt3, and FGF2 and suppressed the BMP signal, thereby reflecting its central role in maintenance of hPSC pluripotency and self-renewal [40]. Interestingly, however, Activin A has paradoxical effects on hPSCs in both maintenance of pluripotency and induction of differentiation, as Activin A-induced differentiation of hPSCs into mesoendodermal cells is well documented, wherein studies suggest that higher concentrations induce differentiation while lower concentrations are necessary for pluripotency [86, 93].

Another member of the TGF*β* superfamily, BMP4, which is known to synergize with leukemia inhibitory factor (LIF) to maintain pluripotency in mESCs, has an opposite role in hPSCs. While BMP signaling maintains self-renewal in mESCs, it induces differentiation in hPSCs [94]. This was revealed through reports which showed that hESCs, grown in serum-free media supplemented with FGF2, were induced by BMP4 to differentiate into a different extraembryonic lineage, the trophoblast [95], or spontaneously differentiate into extraembryonic endoderm-like cells due to BMP2 [96]. This was confirmed by other studies in which the addition of BMP4 in the medium induced a rapid loss of pluripotency in hESCs [92], and the BMP4 antagonist Noggin (500 ng/ml) had a synergistic effect with FGF2 (40 ng/ml) in maintaining the undifferentiated state of hESCs [67, 97]. The latter group showed that BMP2 and BMP4 proteins were detected at higher levels in serum replacement-based hESC cultures, as compared to MEF-conditioned media-based cultures, which could be repressed by either a combination of Noggin and FGF2 or a high dose of FGF2 (100 ng/ml), both of which sustained long-term undifferentiated proliferation of hPSCs in feeder-free culture condition [67]. Xu et al. also showed that both TGF*β* and FGF signalings synergize to antagonize BMP signaling, thereby sustaining expression of genes associated with pluripotency, such as NANOG, OCT4, and SOX2 [91]. Dorsomorphin, another small molecule included in some xeno-free media compositions, acts by inhibiting BMP signaling thereby promoting hPSC self-renewal and preventing BMP-induced differentiation in hPSCs [98, 99].

### 4.4. Wnt Family

Wnt signaling has been implicated in numerous functions in stem cells, and in general, they act to maintain stem cells in their undifferentiated state. Sato et al. showed that the activation of the canonical Wnt pathway was sufficient to maintain the self-renewal capacity of hESCs [71]. They reported that Wnt activation by inhibition of glycogen synthase kinase-3 (GSK-3) or addition of recombinant Wnt3a in the media promoted the growth of compact, undifferentiated hESC colonies, compared to nontreated cells. Furthermore, withdrawal of the GSK-3 inhibitor led to reversal of the pluripotent state and induced differentiation [71]. Dravid et al. on the other hand, reported that Wnt activation was not sufficient to maintain the pluripotent state of hESCs, but Wnt3a application stimulated hESC proliferation instead [100]. Cai et al. also showed that if FGF2 or MEF-conditioned media- (MEF-CM-) derived factors were absent, addition of Wnt3a or Wnt1 stimulated hESC proliferation and also differentiation [101]. In the presence of FGF2 and MEF-CM, however, Wnt can stimulate proliferation of the undifferentiated hESCs [101]. A meta-analysis of microarray data identified the Wnt receptor FZD7 to be a hESC-specific antigen, which upon knockdown led to a rapid change in morphology of cells and loss of expression of pluripotency markers, thereby suggesting the contribution of Wnt signaling to the pluripotency and self-renewal capacity of hPSCs [102]. Wnt activation in media can be achieved by using chemical GSK-3 inhibitors, which are, in increasing order of specificity, lithium chloride, 6-bromoindirubin-3′-oxime (BIO), and CHIR99021, thus eliminating the need for any xenogenic compounds for modulating Wnt signaling [103, 104]. In another study, Hasegawa et al. identified a small molecule Wnt signaling modulator, ID-8, which along with Wnt3a completely prevented Wnt-induced differentiation without affecting proliferation and while maintaining stable hPSC survival as well [105]. Therefore, although the role of Wnt/*β*-catenin signaling in hPSCs is unclear and there is no consensus regarding its effect on self-renewal or differentiation, the above studies highlight how FGF2, Wnt, and TGF*β* signalings collaborate to maintain hPSC self-renewal, pluripotency, and proliferation *in vitro*. In another study, it was reported that Wnt3a and FGF alone were not capable of supporting hPSC growth on feeder-free systems, but with the addition of insulin, transferrin, albumin, chemically defined cholesterol, and a proliferation-inducing ligand (April)/B cell-activating factor belonging to TNF (BAFF), a modified medium named HESCO was developed which was able to support hESC proliferation and self-renewal for over three passages [42]. Interestingly, Tsutsui et al. developed a defined culture system wherein a unique combination of molecules including bFGF, along with inhibitors against GSK, MEK, and Rho-associated kinases (ROCK), allowed long-term maintenance of hESCs through single cell passaging [106].

Recent studies have shown that Wnt/*β*-catenin signaling is active in the naïve state of pluripotency in hPSCs, while it is reduced or absent in the primed state. Naïve hPSCs secrete Wnts that activate Wnt/*β*-catenin signaling in an autocrine or paracrine manner, which promote efficient self-renewal and inhibit their transition to a primed state [107]. Inhibition of Wnt/b-catenin signaling in naïve hPSCs does not lead to differentiation, and the cells continue to express stem cell markers. Also, it induces transition towards primed like hPSC state [107].

## 5. Chemical Compounds

Over the past two decades, majority of hPSCs have been maintained in culture including xenobiotic components which carry a risk of contamination of immunogens or pathogens rendering those cells incompetent for regenerative medicine applications. Hence, the development of chemically defined or xeno-free systems for the hPSC culture system is necessary. To circumvent these challenges researchers have been putting efforts to develop defined conditions for hPSCs by using chemically synthesized molecules which regulate a biological process. Chemical compounds such as MEK inhibitors (PD98059 and PD0325901), GSK-3 inhibitors (Wnt signal activator) (BIO and CHIR99021), Rho-associated kinase (ROCK) inhibitor (Y-27632), FGF/RTK inhibitor (PD173074), and TGF*β*/ALK inhibitor (SB431542) are commercially available and most widely used for the maintenance, proliferation, and differentiation of hPSCs. Since FGFs/RTKs, TGF*β*s/ALKs, and Wnts/GSK-3 signaling have been described in previous chapters, compounds widely used for regulating other signaling in hPSCs will be reviewed in this chapter.

### 5.1. ROCK Inhibitor

Rho-associated protein kinase (ROCK), a downstream effector of Ras homolog gene family member A (RhoA), is a kinase belonging to the family of serine/threonine kinases and is involved mainly in regulating the shape and movement of cells by acting on the cytoskeleton [108]. Poor viability of hPSCs during passage caused by apoptosis, due to actomyosin hyperactivation called blebbing [109], is an obstacle to researchers, hampering daily routine culture such as dissociation/expansion. ROCK inhibitors such as Y-27632 reduce this blebbing and minimize apoptosis, thereby enhancing the cloning efficiency after cell dissociation [110]. Studies have also shown that the ROCK inhibitor enhances the efficiency and survivability of hPSCs after freeze-thaw cycles [111, 112]. It also helps to keep hPSCs in an undifferentiated state in feeder as well as feeder-free conditions [113]. In the absence of ROCK inhibition, apoptosis can be reduced by dissociating hPSCs as cell clumps instead of single cells, while ROCK inhibitor addition at the time of passage can partially reduce the apoptosis induced during single cell dissociation. Therefore, further studies highlighting methods for reducing stress induced by dissociation into single cells even further are required for achieving maximum viability.

### 5.2. Compounds Used for Naïve Culture Condition

Initial attempts to derive or maintain naïve hESCs relied on the continuous ectopic expression of pluripotency genes in addition to hPSC media containing leukemia inhibitory factor (LIF) and inhibitors of both MEK and GSK-3 (termed 2i) [114–116]. However, recently, Gafni et al. developed a chemically defined media called naïve human stem cell medium (NHSM), which contained a basal media of DMEM + KSR, N2, AlbuMAX and insulin, and growth factors FGF2 and TGF*β*, supplemented with 2i/LIF (described above), p38/MAPK inhibitor (SB203580), and the noncanonical Wnt/c-Jun-N-terminal kinase (JNK) inhibitor (SP600125), which was further optimized to improve cell viability by addition of the ROCK inhibitor (Y-27632) and protein kinase C (PKC) inhibitor (Gö6983) [13]. Other studies have shown, however, that minimal media consisting of bFGF, with MEK and GSK inhibitors [15], or additionally with the ROCK inhibitor and LIF [117] can support naïve pluripotency. Interestingly, [118] showed that a different cocktail of 5 inhibitors (5i) including inhibitors of MEK (PD98059), GSK (IM12), ROCK (Y-27632), BRAF (SB590885), and LCK/SRC (WH-4-023) along with LIF, bFGF, and Activin A (5i/L/F/A) was the most efficient for derivation and maintenance of naïve hPSCs. They also showed that inclusion of 20% KSR was detrimental to induction of naïve pluripotency, compared with culture in N2/B27 media, as well as supplementation with the GSK inhibitor (CHIR99021) was tricky because lower concentrations improved the maintenance of naïve pluripotency, while higher concentrations led to the opposite. The reduction in CHIR99021 concentration to 1 *μ*M also cooperated well with MEK and PKC inhibitions, in the presence of LIF (termed t2iL + Go) for maintaining naïve pluripotency [119]. Interestingly, a recent report has shown that Wnt5A is a crucial component which, together with 2i/LIF and bFGF, promotes induction of naïve pluripotency in established hESC lines [120].

## 6. Extracellular Matrices

The knowledge of feeders and an understanding of the crosstalk between ECM components, cells, and media have been exploited to develop feeder-free extracellular matrices (ECMs) which mimic the conditions that feeders provided, for hPSCs while reducing the dependency on xenogeneic components. As mentioned earlier, one of the first substrates used as a feeder-free alternative was Matrigel, which is mainly composed of attachment proteins like laminin, entactin, collagen IV, and heparan sulfate proteoglycans, in addition to growth factors, and each of these individual components show varying levels of efficiency in supporting hPSC culture [36]. However, since Matrigel is derived from an animal source, it can introduce unwanted xenogeneic contaminants too, thus making this unsuitable for clinical therapies. Significant progress has been made in identifying individual ECM components such as laminin-511, fibronectin, and vitronectin in the hope of developing chemically defined and synthetic substrates. Laminin-coated surfaces are very efficient in supporting the pluripotency and proliferation of hPSCs, while collagen IV and fibronectin are not [36]. Furthermore, specific laminin isoforms show different effects as ECM substrate with isoforms -111, -332, and -511, but not -211 and -411, being able to support the attachment and proliferation of undifferentiated hPSCs. In addition, it has been shown that supportive feeder cells and hPSCs produce laminin isoforms -511/-521 and express the integrin *α*6*β*1 receptor, the primary receptor for these laminin isoforms [121]. Since integrins are the principal molecules that mediate cell-ECM interactions, other substrates such as vitronectin, which has been shown to support hPSC self-renewal via integrin *α*V*β*519, have been developed [122]. Recombinant human laminin-511 and -521, vitronectin and E-cadherin, and their short fragments were amongst the first defined ECM substrates to be described, and they have now been routinely employed in feeder-free culture. Their use therefore represents an important milestone in hPSC culture. These led to the development of polymers, modified with synthetic peptides, such as SyntheMax [123]. However, the exact mechanisms of cell-ECM and cell-cell adhesion in hPSCs are still not clear and need to be addressed.

## 7. hPSC Bulk Culture Systems

For applications in engineering and medicine, hPSCs need to be generated in quantities of 10^10^ cells or more, while also adhering to cGMP guidelines and the requirements of regulations governing hPSC-based therapeutics [124, 125]. 2D adherent feeder-free culture can be “scaled-out” by multiplying the culture volume through the use of multilayered flasks, and some robotic platforms that can automate the process have been shown to provide large-scale quality-controlled manufacture of hPSCs [126, 127]. However, this method of bulk culture is still limited for commercial use by requirements of considerable space, time, cost, and operators, while also restricting online monitoring of culture parameters. This can considerably affect the reproducibility and stability of the cells in culture. Three dimensional (3D) or suspension culture in spinner flasks or Erlenmeyer flasks has been reported for bulk expansion of hPSCs [128–131], while their volume, control of culture parameters, and monitoring system may not be sufficient for hPSC applications that require large-scale production of hPSCs. Thus, bioreactors providing control over the culture environment and real-time monitoring of the system parameters need to be developed for industrial level expansion of hPSCs. In these 3D suspension culture systems, cells are cultured in the form of matrix-free hPSC clumps as aggregates or as hPSCs immobilized on microcarriers or by microencapsulation of the hPSCs. Importantly, the media used for such large-scale 3D expansion of hPSCs are based on the culture media used in 2D feeder-free systems itself, such as KSR-based media, mTeSR1, StemPro hESC SFM, and Essential 8. Defined protocols for hPSC suspension culture in mTeSR1 medium have been published and have been widely used [132, 133]. However, hPSCs cultured successfully in 2D systems in certain media may not continue to behave in the same way when cultured in the same media, in 3D suspension. This could be because certain media components may not remain stable in suspension, as shown by a recent study, where insulin was found to precipitate from commercial hPSC media (E8, TeSR-E8, mTeSR1, and StemMAXs iPS-Brew XF), only when used in a peristaltic pump circuit suspension system [62]. Interestingly, the depletion of insulin from culture media led to excessive disintegration of hPSC aggregates with subsequent loss of viability in the applied culture system, while the omission of bFGF, TGF*β*1, or transferrin did not significantly hamper the morphology and viability of hPSCs. This showed how insulin was an absolute necessity for hPSC maintenance [62]. Studies such as these highlight how necessary it is to closely monitor media components while establishing automated bulk culture systems. In an interesting report by Lipsitz et al., it has been shown that the conversion of hPSCs from a primed to an alternative naïve-like state is advantageous for suspension-grown hPSCs and shifts them to a high-yield state [134]. They reported that for dynamic suspension cultures, the use of media supplemented with LIF, TGF*β*1, and FGF2, including inhibitors of GSK-3 (CHIR99021), JNK (SP600125), p38/MAPK (BIRB796), and PKC (Gö6983), was the most efficient in supporting a faster growth rate and higher densities, compared to conventional hPSC media. Furthermore, they showed that the conversion of primed hPSCs to this alternative “high-suspension-yield” state in adherent culture required the MEK inhibitor (PD0325901) in addition to the above 4 inhibitors, following which when cells were transferred to suspension culture, it was essential to withdraw MEK/ERK inhibition, in order to maintain pluripotency. This shows how our understanding of the role of media components at each stage of the process can enable more efficient and cost-effective manufacturing of hPSCs for therapeutic applications. Overall, this system demonstrated how cell-state conversion induced by specific media components provided a strategy for long-term, high-density expansion of hPSCs, in scalable suspension cultures. This emphasizes the importance of culture media optimization to overcome the challenge of the low yield of hPSCs in suspension bioreactors [134].

These 3D culture systems usually consist of biomaterials to mimic the *in vivo* microenvironment and 3D niche much better than 2D systems. These biomaterials contribute to signaling within the culture and improve cellular crosstalk, thereby simulating the biophysical and biochemical properties of the native cellular niche. Therefore, such culture systems can be used to study embryogenesis and organogenesis as well as for drug toxicity and screening assays. It is worth noting that such scalable hPSC suspension cultures can also be used as a convenient starting point for inducing them to differentiate by changing hPSC expansion media to lineage-specific media [135–137]. Thus, 3D cultivation of hPSCs in bioreactors allows mass and automated production of hPSCs and their differentiated cells. Indeed, recent developments in stem cell research have led to the establishment of protocols using which hPSCs can be differentiated in suspension to various cell types and hence grown into miniorgans, termed “organoids.” Organoid cultures are structures composed of thousands of cells, often coming from different lineages (that make up an organ *in vivo*), which self-assemble themselves into 3D structures, in the presence or absence of exogenous ECM substrates. This creates a dynamic microenvironment between the self-renewing and differentiating cells, providing the closest *in vitro* equivalent of the architecture of real organs. They exhibit increased functionality compared to 2D-differentiated cells and thus have wide-ranging applications in research and the clinic [138].

Various vessels and bioreactors are available for the hPSC scale-up culture system, such as the stirred tank, airlift, spinner flasks, wave-rocking, rotating wall, hollow fiber, multiplate, magnetic microcarrier, and 3D scaffold bioreactor. They consist of a glass or plastic vessel equipped with or without an impeller that ensures a homogenous growth environment by efficient mixing of the cells, nutrients, and gases, while maintaining an uplift against gravity, to keep cell aggregates or microcarriers in suspension [139, 140]. hPSCs can be inoculated as single cells or aggregates, with single cell inoculation, using the ROCK inhibitor, being better at preventing extensively large-sized aggregates that can limit nutrient diffusion [141]. Nutrient feeding can be carried out in batches or by perfusion, although the latter is reported to result in a more uniform environment enabling superior cell densities and yields [142]. The key parameters such as pH, dissolved oxygen (DO) levels, fluid shear stress, growth factors, nutrients, and metabolite concentrations in hPSC scale-up culture are precisely and carefully controlled to ensure a uniform environment with adequate nutrient levels and oxygenation [129].

Using coated microcarrier beads in bioreactors can be considered as a practical option. Several studies have demonstrated that using microcarriers such as polystyrene coated with laminin and vitronectin, vitronectin and HSA further treated with UV, or those that are positively charged with cellulose achieve high attachment efficiencies and viability even at the high confluency, while also reducing consumption of media and growth factors owing to their adjustable growth surface area [143–146]. Beads with multiple pores give a much larger surface area for cell adhesion and gas diffusion, than flat 2D culture, while the adherent hPSCs on/in the beads may be very similar to what is obtained in traditional 2D culture [147]. Lastly, microencapsulation of hPSCs in hydrogels, such as calcium alginate capsules, has been developed as a technique to minimize excessive clumping in suspension culture, as well as improve cell recovery rates after cryopreservation [148]. Although this method offers increased cell protection, it is limited by reduced diffusion of gases and nutrients through the capsule, plus the requirement of decapsulation for cell harvest. Thus, 3D cell culture systems can offer substantial benefits for hPSC culture and are an attractive platform for manufacture of cellular products, due to their scalability, ease of monitoring, and convenient cell feeding and harvesting options. However, certain important issues, such as controlling growth rate, aggregate size, differentiation pressure, and ensuring genomic stability, need to be addressed before they can be approved for clinical applications [149].

## 8. Conclusion

The growth and advancement in the applications of hESC/hiPSC technology have been accompanied by a huge body of work dedicated to optimizing and standardizing hPSC culture. Optimization of culture systems is not only limited to defining culture media components but also the extracellular matrices, environmental cues, and modes of passaging. As discussed above, this has led to the development of simple and defined culture conditions and also facilitated the development of 3D and bulk cell culture systems. Chemically defined media are ideal for minimizing lot-to-lot variation and ensure consistency, for both research-based and clinical applications, while xeno-free and cGMP-compliant culture systems are preferred for cellular transplantation. Efforts in this direction have largely focused on understanding the signaling molecules required, replacing nonhuman components of media and developing synthetic substrates, which have an immediate application in disease modelling and can eventually be used to engineer cells and their substrates for therapeutic applications.

The recent findings about the isolation and maintenance of naïve hPSCs have given immense insight into early human development, but it is yet to be determined how relevant these different states of pluripotency are, with respect to downstream engineering applications in research and therapy. As such, future platforms should ideally attempt to be adaptable for the bioprocessing of both naïve and primed hPSCs.

With stem cell therapies becoming a reality, through the approval of human trials, preventing any alterations in the cellular state and identity is of paramount importance. As new therapies emerge, questions about the safety and efficacy of the cellular products will also rise. Studies have revealed the heterogeneity of hPSCs in culture, and some procedures to isolate and propagate homogenous hPSC clones have been described. Nevertheless, clearer and more stringent regulations need to define how to maintain cellular homogeneity in scalable culture systems. It is important to note that one of the obstacles in the implantation of hPSC-based therapies is the risk of tumorigenicity, owing to the intrinsic properties of unlimited self-renewal and expansion of hPSCs. However, in addition to the in vivo effects, prolonged periods of *in vitro* expansion required for cell therapy itself can lead to stochastic generation of chromosomal aberrations which can then accumulate rapidly through positive selection pressure. Such culture adaptations by cells precede the highly undesirable build-up of gross genomic abnormalities, characteristic of certain high passage number hPSC lines. Therefore, along with the establishment of bulk culture systems, the validation of assays that can efficiently and reproducibly monitor growth conditions, cellular stress, spontaneous differentiation signals, gene expression profiles, and chromosomal integrity during production, processing, and storage of hPSCs is absolutely vital to the success of all downstream applications. Future research should address these challenges by establishing protocols for providing robust, stable, and homogenous cell populations as raw material.

Another potential challenge lies in determining the long-term effects of major media used for propagation of hPSCs in large-scale culture systems and addressing the costs associated with maintenance of these systems. The economics of hPSC processing are as important to address, as are the concerns with their safety and functionality in patients. Costs associated with media components have been largely reduced with an improved understanding of their functions and the subsequent discovery of small molecules which can substitute for the expensive growth factors and cytokines, as discussed above. Apart from making media cost-effective, it has also led to the media being more defined, hence increasing their reproducibility.

There are now multidisciplinary approaches available for the derivation and culture of different stages of pluripotency, development of xeno-free culture conditions as well as the generation of GMP-compatible protocols, which would help standardize and streamline the process on a commercial scale. The amalgamation of 3D culture systems, chemically defined media, and synthetic biomaterials mimicking ECMs shows enormous potential in improving propagation, safety, and functionality of stem cells for various applications.

The derivation and maintenance of the raw material, hPSCs, including both hESCs and hiPSCs, have to be done in a manner that recapitulates their equivalent niche in vivo; therefore, the media in which these cells are cultured in are central to the success of all downstream applications. Therefore, as a starting point, it is necessary to understand the media components and their roles in regulating the self-renewal, proliferation, survival, stability, and functionality of hPSCs as this is a critical step in ensuring large-scale, safe, reproducible, and quality-controlled expansion of hPSCs for use in stem cell engineering.

## Figures and Tables

**Table 1:** Commonly used commercial media for feeder-free culture of hPSCs.

Medium	Components	Extracellular matrix	XF/CD	Company
mTeSR™1	DMEM/F12, BSA bFGF, TGF*β*, insulin, transferrin, cholesterol, lipids, pipecolic acid, GABA, *β*-mercaptoethanol	Corning® Matrigel®, vitronectin	NA	STEMCELL Technologies
TeSR™2	DMEM/F12, with recombinant HSA, bFGF, TGF*β*, insulin, transferrin, cholesterol, lipids, pipecolic acid, GABA, *β*-mercaptoethanol	Corning® Matrigel®, vitronectin	XF, CD	STEMCELL Technologies
Essential 8™	DMEM/F12 bFGF, TGF*β*, insulin, transferrin, selenium, ascorbic acid	Corning® Matrigel®, vitronectin	XF, CD	Thermo Fisher Scientific
TeSR™-E8™	Based on E8 formulation	Corning® Matrigel®, vitronectin	XF, CD	STEMCELL Technologies
StemPro®	DMEM/F12, BSA bFGF, TGF*β*, Activin, transferrin, LR3-IGF1, HRG1*β*	Geltrex®	NA	Thermo Fisher Scientific
PluriSTEM™	DMEM/F12, HSA Activin A, TGF*β*1, bFGF, lipids, insulin, transferrin, selenium	Not defined	XF	Millipore

XF: xeno-free; CD: chemically defined medium; NA: not available (neither XF nor CD).
